# Heptadecanoic Acid Is Not a Key Mediator in the Prevention of Diet-Induced Hepatic Steatosis and Insulin Resistance in Mice

**DOI:** 10.3390/nu15092052

**Published:** 2023-04-24

**Authors:** Christopher A. Bishop, Tina Machate, Janin Henkel, Matthias B. Schulze, Susanne Klaus, Karolin Piepelow

**Affiliations:** 1 Department Physiology of Energy Metabolism, German Institute of Human Nutrition Potsdam-Rehbruecke (DIfE), 14558 Nuthetal, Germany; christopher_allen.bishop@dife.de (C.A.B.); karolin.piepelow@dife.de (K.P.); 2 Department of Nutritional Biochemistry, Faculty of Life Sciences: Food, Nutrition and Health, University of Bayreuth, 95326 Kulmbach, Germany; 3 Institute of Nutritional Science, University of Potsdam, 14469 Potsdam, Germany; 4 Department Molecular Epidemiology, German Institute of Human Nutrition Potsdam-Rehbruecke (DIfE), 14558 Nuthetal, Germany

**Keywords:** OCFA, high-fat diet, pentadecanoic acid, heptadecanoic acid, dairy fat, liver inflammation, hepatic lipid accumulation, lipogenesis, insulin resistance, primary mouse hepatocytes

## Abstract

Epidemiological studies found that the intake of dairy products is associated with an increased amount of circulating odd-chain fatty acids (OCFA, C15:0 and C17:0) in humans and further indicate that especially C17:0 is associated with a lower incidence of type 2 diabetes. However, causal relationships are not elucidated. To provide a mechanistic link, mice were fed high-fat (HF) diets supplemented with either milk fat or C17:0 for 20 weeks. Cultured primary mouse hepatocytes were used to distinguish differential effects mediated by C15:0 or C17:0. Despite an induction of OCFA after both dietary interventions, neither long-term milk fat intake nor C17:0 supplementation improved diet-induced hepatic lipid accumulation and insulin resistance in mice. HF feeding with milk fat actually deteriorates liver inflammation. Treatment of primary hepatocytes with C15:0 and C17:0 suppressed JAK2/STAT3 signaling, but only C15:0 enhanced insulin-stimulated phosphorylation of AKT. Overall, the data indicate that the intake of milk fat and C17:0 do not mediate health benefits, whereas C15:0 might be promising in further studies.

## 1. Introduction

In recent years, the role of circulating odd-chain fatty acids (OCFA) in human health has been highlighted, as several observational studies have described an inverse association with the incidence of non-alcoholic fatty liver disease [1,2], insulin resistance and type 2 diabetes [3,4,5,6,7], but investigations concerning a mechanistic link are lacking. The majority of research only focuses on the role of OCFA as a biomarker for dietary food intake assessment [8]. However, a number of studies show that multiple nutritional factors influence circulating concentrations of OCFA. Data provide evidence that plasma OCFA are affected by dietary fiber intake, dairy product intake, fish consumption [9,10,11,12] and by the intestinal microbial composition [13]. Thus, the use of OCFA as a biomarker for food intake should be strongly reconsidered.

The most significant OCFA in human plasma are pentadecanoic acid (C15:0) and heptadecanoic acid (C17:0). In many studies, both OCFA are collectively referred to have positive health outcomes even though data show that usually one fatty acid has a stronger effect on a specific outcome. Plasma levels of C15:0 seem to reflect the intake of dairy products, and most studies support a weaker positive association of this fatty acid with insulin sensitivity, compared to C17:0 [4,14,15,16]. Nevertheless, other studies have demonstrated that C15:0 can exert anti-cancer effects in breast [17,18], lung, pancreatic, or liver cancer cells in vivo [19] and promote an anti-inflammatory state in obese mice [20]. In contrast, a high dietary fiber diet mainly results in an increase in C17:0 [9], which is rather linked with an improvement in insulin resistance [21,22]. Xu et al. already demonstrated that glucose import and PI3K/Akt signaling are suppressed by C17:0 in lung adenocarcinoma cells [23]. However, investigations considering a mechanistic link of endogenous OCFA in hepatic health are lacking. Especially, the effect of C17:0 on liver metabolism, the main organ of OCFA synthesis, has remained elusive. Thus, it is important to consider C15:0 and C17:0 individually in their function and to further elucidate their possible impact on hepatic fat and insulin sensitivity.

As dairy fats are an exogenous source of OCFA, it is one of the components that has attracted more attention in recent years; however, the importance of dairy fat in metabolic health is controversially discussed [24,25]. Further, it should be taken into account that a high exogenous OCFA intake could also contribute to propionyl-CoA formation because they can be oxidized in the same manner as even-numbered fatty acids, but the successive removal of two carbon units results in the formation of propionyl-CoA instead of acetyl-CoA [26]. Recently, we demonstrated that propionate is apparently used for the hepatic synthesis of OCFA in mice and humans by using propionyl-CoA as a primer for fatty acid synthase [9,22]. Both gut-derived propionate (by inulin feeding) as well as dietary supplementation of propionate led to an improvement in high-fat-diet-induced hepatic steatosis and insulin resistance [21]. As propionate is a relevant source of circulating OCFAs, the above-mentioned beneficial association of plasma OCFAs and a decreased type 2 diabetes risk could be attributed to an increased availability of propionyl-CoA. Nevertheless, the regulatory mediator that governs the development of hepatic steatosis and insulin resistance has not yet been clarified. For this reason, the aim of this study was to investigate if heptadecanoic acid (as an OCFA) is able to improve diet-induced metabolic alterations and further to examine the role of milk fat as a source rich in OCFA.

## 2. Materials and Methods

### 2.1. Animals and Experimental Set Up

Animal experiments were approved by the ethics committee of the Ministry for Environment, Health, and Consumer Protection of Brandenburg, Germany (approval no. 2347-17-2018). Male C57BL/6JRj mice (8 weeks of age) were purchased from Janvier Labs (Le Genest-Saint-Isle, France). Animals were group housed in polycarbonate cages in a climate-controlled room (22 ± 2 °C, relative air humidity 55 ± 5%) with a 12 h light:dark cycle and ad libitum access to food and water. After two weeks of adaptation on standard chow diet (Altromin fortified type 1314, Altromin GmbH, Lage, Germany), mice were switched to semi-synthetic experimental diets (Table S1). Animals were either fed a low-fat diet (LFD, n = 12), high-fat diet (HFD, n = 12) or HFD supplemented with either 14% of milk fat (HFMF, Uelzena eG, Uelzen, Germany, n = 12), 5% of heptadecanoic acid (HFC17, Merck, n = 12) or with 5% propionate (HFPr, Merck KGaA, Darmstadt, Germany, n = 12). After 20 weeks of dietary intervention, 2 h fasted animals (6–8 am) were euthanized with isoflurane, and peripheral blood was taken by cardiac puncture. Tissues (liver, kidney, epididymal WAT, subcutaneous WAT, quadriceps muscle, gastrocnemius muscle and brown adipose tissue) were weighed and frozen in liquid nitrogen before they were stored at −80 °C. One piece of the liver and the whole pancreas were embedded in paraffin for immunohistochemistry.

### 2.2. Metabolic Phenotyping

Body weight was measured every 2 weeks on a scale. Body fat was determined by a nuclear magnetic resonance spectrometer from EchoMRI™ (Echo Medical Systems, Houston, TX, USA). Indirect calorimetry was performed in week 12 of intervention with individually caged mice using the Pheno Master System (TSE Systems GmbH, Bad Homburg, Germany).

### 2.3. Tolerance Tests

Oral glucose tolerance tests (OGTT) were performed in week 16 of intervention after a fasting period of 16 h (4 p.m.–8 a.m.) with one half of the animals (n = 6). Glucose (2 g/kg body weight) was administered orally, and blood glucose was measured after 0, 15, 30, 60, 120, 180 and 240 min from tail vein with a glucometer. Plasma insulin concentrations were measured after 0, 15, 30 and 60 by ELISA (ALPCO). Insulin tolerance tests (ITT) were performed in week 16 of intervention after 2 h of fasting (8–10 am) with the other half of the animals (n = 6). Insulin (0.75 U/kg body weight) was injected intraperitoneally (i.p.), and blood glucose was measured after 0, 15, 30, 60 and 120 min from tail vein with a glucometer.

### 2.4. Immunohistochemistry

Pancreatic sections (2 µm) were de-paraffinized and rehydrated in Roti-Histol (Carl Roth, Karlsruhe, Germany) and a decreasing serial solution of ethanol. Slides were heated in citrate buffer (10 mM citrate acid, 0.05% Tween 20) in a pressure cooker using a microwave for 30 min at 900 W followed by a cooling step (30 min, RT). Sections were incubated with blocking solution (5% BSA/TBST, 1 h, RT), followed by primary antibodies (1 h, RT). Rabbit anti-insulin (Abcam, Cambridge, UK, ab181547) was used as primary antibody. Hydrogen peroxide blocking was performed with a 0.3% solution (Carl Roth) for 15 min at RT, followed by incubation with HRP-labeled Goat Anti-Rabbit IgG (1 h, RT) (Abcam, ab205718). Before mounting with VectaMount Mounting Medium (H-5000), tissue sections were incubated with 3,3’-diaminobenzidine in chromogen solution (ImPACT DAB Peroxidase Substrate Kit, Vector Laboratories, Burlingame, CA, USA) and counterstained with hematoxylin (Carl Roth). Stained insulin in whole pancreatic sections were imaged with a MIRAX-MIDI Scanner (Carl Zeiss AG, Oberkochen, Germany), equally edited with Pannoramic Viewer and analyzed with ImageJ Software.

### 2.5. Plasma and Tissue Measurements

Plasma triglycerides (TG Determination Kit, Merck, Rahway, NJ, USA), cholesterol (Cholesterol liquicolor, Human GmbH, Wiesbaden, Germany) and non-esterified fatty acids (NEFA C kit, Wako Chemicals Europe GmbH, Neuss, Germany) were measured in 1:2 diluted samples according to the respective protocol. Liver tissue was extracted with 10 mmol/L sodium phosphate buffer (pH 7.4) containing 1 mmol/L EDTA and 1% (*v*/*v*) polyoxyethylene(10)-tridecyl-ether, and TG were measured following the same protocol.

Relative intrahepatic lipid content (IHL) was quantified in random microphotograph liver sections that were stained with H&E [21] and calculated as previously described [27].

### 2.6. Quantitative Real Time PCR (qRT PCR)

RNA was extracted with TriFast reagent (PeqLab, Erlangen, Germany), followed by DNase treatment (DNase I, RNase-free, Thermo Scientific, Waltham, MA, USA) and cDNA synthesis (LunaScript RT SuperMix Kit, NEB, Ipswich, MA, USA) according to the respective manual. qRT-PCR was performed as previously described [22] with the modification that Luna Universal qPCR Master Mix (NEB) was used. Primer sequences are listed in Table S2. Gene expression was calculated as ddCT, using the reference gene *B2m*. Data were expressed relative to the LFD group, which was normalized to a value of 1.

### 2.7. Western Blot

Protein was isolated using RIPA-buffer containing Halt Protease and Phosphatase Inhibitor Cocktail (Thermo Fisher Scientific, Waltham, MA, USA). Immunoblotting and detection were performed as already described [22,28]. Primary antibodies were used as follows: CD36 (#MAB2519), ACSS2 (#PA5-26612), ACSS3 (#16204-1-AP), FASN (#3189), ELOVL6 (#21160-1-AP), SCD1 (#sc-515844), HACL1 (#HPA035496), pAKT/AKT (#9271/#9272), pS6K/S6K (#sc-8416/#sc-8418), pJAK2/JAK2 (#3776/#3230), pSTAT3/STAT3 (#9131/#9139). Protein expression was normalized to ponceau staining or GAPDH (#AM4300).

### 2.8. Long-Chain Fatty Acid (LCFA) Analysis

LCFA composition (C14:0; C15:0; C16:0; C16:1n7c; C17:0; C18:0; C18:1n9c; C18:1n7c; C18:2n6c; C20:0; C18:3n3; C20:1n9; C20:2n6; C20:3n6; C20:4n6; C20:5n3; C24:0; C22:4n6; C22:5n6; C22:5n3; and C22:6n3) in phospholipid fraction was determined in diets, liver tissue, plasma and epididymal white adipose tissue (eWAT) by gas chromatography as already described [29]. Liver enzyme activity indices were calculated as previously described [30].

### 2.9. Primary Hepatocytes

Primary hepatocytes were isolated from livers of 10–14-week-old male C57BL/6JRj mice as previously described [31]. Cells were treated with either pentadecanoic acid (125 µM C15:0, Merck) or heptadecanoic acid (125µM C17:0, Merck) in the presence of complexed BSA:palmitic acid (125 µM BSA: 250 µM PA) for 24 or 48 h in serum starvation media. PA concentration was chosen based on literature data [32,33]. For treatments, half of the PA concentration was replaced by the respective FA to prevent lipotoxicity effects. For insulin stimulation, media were changed to insulin-free media, 2 h prior to stimulation (15 min) with 100 nM insulin.

### 2.10. Statistical Analysis

GraphPad Prism 9 (GraphPad Software, La Jolla, CA, USA) was used for statistical analysis. All data are represented as mean and the standard error of mean. Kolmogorov–Smirnov test was used for normal distribution and homogeneity of variances. Data of HFD vs. HFMF vs. HFC17 (or PA vs. PA-C15 vs. PA-C17) were tested for statistical significance with an ordinary one-way ANOVA using a Bonferroni’s post hoc test (normally distributed data) or Kruskal–Wallis test using Dunn’s post hoc test (non-normally distributed data). Data of HFD vs. HFPr were analyzed by using an unpaired, two-sided t test (normally distributed data) or Mann–Whitney U test (non-normally distributed data). Data of principal component analysis were standardized to have a mean of 0 and a standard deviation of 1. Principle components were selected by parallel analysis, which accounts for variances in the data due to random error or noise and according to the highest eigenvalues (LFD vs. HFD vs. HFMF vs. HFC17: PC1, 8.4; PC2, 4.4; PC3, 3.1; LFD vs. HFD vs. HFPr: PC1, 7.5; PC2, 4.5; PC3, 4.2)

## 3. Results

### 3.1. Long-Term Feeding of Milk Fat and C17:0 Elevates OCFA in Different Compartments

**Figure 1:**
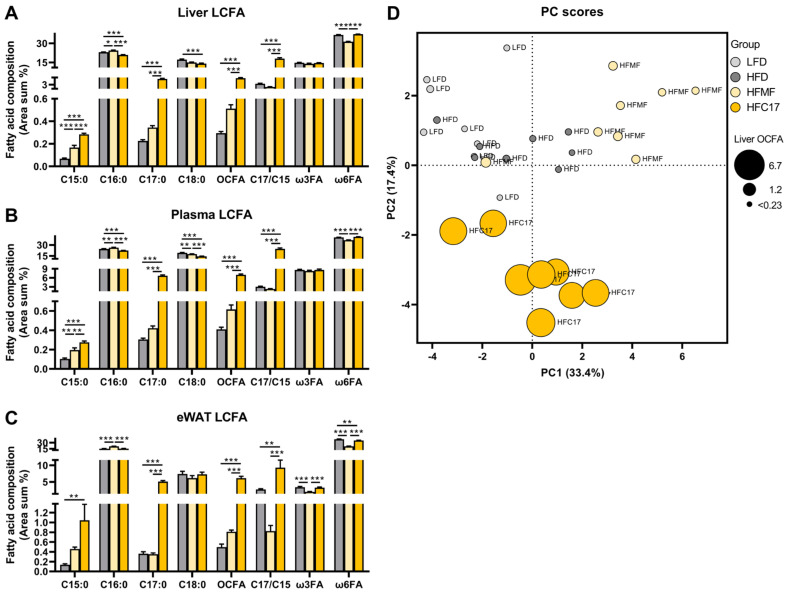
Supplementation of C17:0 leads to the highest induction of OCFA in liver, plasma and white adipose tissue. C57BL/6JRj mice were fed semi-synthetic low-fat diet (LFD), high-fat diet (HFD, depicted in grey) or HFD supplemented with either 14% milk fat (HFMF; depicted in light yellow) or 5% heptadecanoic acid (HFC17, depicted in dark yellow) for 20 weeks. Composition of long-chain fatty acid (LCFA) phospholipids in (**A**) liver, (**B**) plasma and (**C**) epididymal white adipose tissue (eWAT) after 20 weeks of intervention, n = 7−8. Results are expressed as area percentage of each fatty acid relative to the total area of all detected fatty acids. (**D**) Principal component (PC) analysis showing the score plot of data including hepatic OCFA formation. Data are mean + SEM, * *p* < 0.05; ** *p* < 0.01, *** *p* < 0.001. OCFA, odd-chain fatty acids; ω3FA, omega-3 fatty acids; ω6FA, omega-6 fatty acids.

By comparing the long-chain fatty acid profiles of various tissues after feeding OCFA-rich diets, it was apparent that milk fat feeding increases the amount of C15:0 in liver and plasma phospholipids but not in epididymal white adipose tissue (Figure 1A–C). Only C17:0 supplementation led to an enhanced amount of both OCFA (C15:0 and C17:0) in all analyzed tissues. These strong increases were in line with lower amounts of C16:0 and C18:0 in liver and plasma phospholipids. Feeding the HFMF diet, on the other hand, showed higher C16:0 levels, but lower amounts of C18:0 and omega-6 fatty acids in almost all tissues (Figure 1A–C). Calculation of the C17:C15 ratio showed that milk fat intake has a tendency toward a lower ratio (HFD: 3.4 ± 0.2 vs. HFMF: 2.1 ± 0.1), indicating a higher induction of C15:0 (C15:0: 3×; C17:0: 1.5×, compared to HFD), whereas C17:0 supplementation shows a higher ratio, as expected, indicating a higher induction of C17:0 (Figure 1A–C). Principal components analysis (PCA) score plots illustrate the grouping of variables in the data sets. Here, the score plot demonstrates that OCFA levels in the liver, which is the main organ for their endogenous synthesis, were distinct after HFMF and HFC17-feeding, compared to control animals (Figure 1D). Differences were mainly attributed to C17:0-supplemented mice, which showed much higher total OCFA levels. In general, milk fat supplementation showed the lowest formation of OCFA, also compared to propionate supplementation (Figure S1).

### 3.2. Long-Term Supplementation of C17:0 Does Not Improve Diet-Induced Hepatic Steatosis

**Figure 2:**
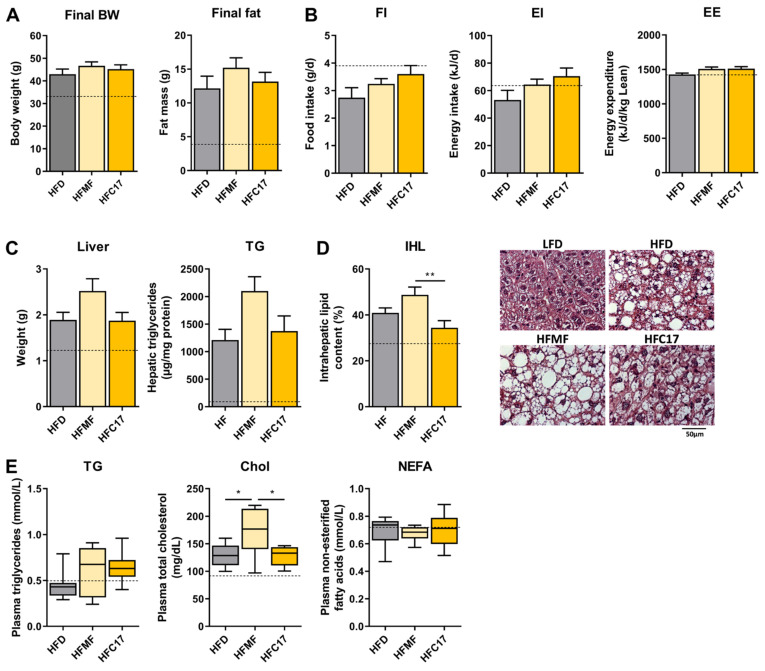
High-fat feeding with C17:0 does not affect hepatic lipid accumulation, whereas milk fat supplementation exacerbates it. C57BL/6JRj mice were fed semi-synthetic low-fat diet (LFD, dotted line), high-fat diet (HFD, depicted in grey) or HFD supplemented with either 14% milk fat (HFMF; depicted in light yellow) or 5% heptadecanoic acid (HFC17, depicted in dark yellow) for 20 weeks. (**A**) Final body weight (BW) and fat mass after 20 weeks of intervention, n = 12. (**B**) Average food intake (FI), energy intake (EI) and energy expenditure (EE), n = 8. (**C**) Liver tissue weight (n = 12), hepatic triglyceride (TG) concentration (n = 8) and (**D**) relative intrahepatic lipid content (IHL) of H&E-stained liver sections with representative images, n = 8. (**E**) Circulating concentrations of TG, cholesterol (Chol) and non-esterified fatty acids (NEFA) after 20 weeks of intervention, n = 8. Data are mean +/− SEM, * *p* < 0.05; ** *p* < 0.01.

Supplementation of both, C17:0, or MF appeared to have no differential effect on body weight gain or body fat accumulation (Figure 2A). This is in line with the fact that neither energy intake nor energy expenditure was affected (Figure 2B). Dietary milk fat intake tends to have a higher hepatic triglyceride concentration (*p* = 0.13) (Figure 2C) and shows larger intrahepatic lipid droplets (Figure 2D), compared to HF control. Notably, cholesterol levels in the circulation are increased after MF supplementation, which is reflective of their high concentrations in milk fat (Figure 2E). Overall, long-term dietary intake of milk fat or C17:0 does not have a major impact on the metabolic phenotype in mice under HF conditions.

### 3.3. High-Fat C17:0 Supplementation Does Not Enhance Insulin Sensitivity

**Figure 3:**
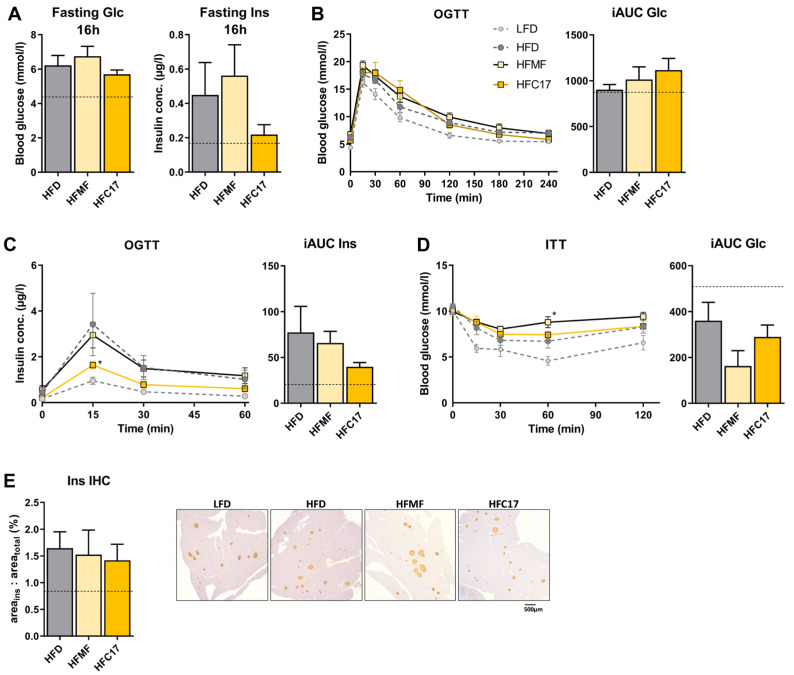
Long-term supplementation of C17:0 elicits negligible effects on insulin sensitivity. C57BL/6JRj mice were fed semi-synthetic low-fat diet (LFD, dotted line), high-fat diet (HFD, depicted in grey) or HFD supplemented with either 14% milk fat (HFMF; depicted in light yellow) or 5% heptadecanoic acid (HFC17, depicted in dark yellow) for 20 weeks. (**A**) Glucose (Glc) and insulin (Ins) concentrations after 16 h of fasting, n = 6. (**B**) Time-dependent blood glucose (BG) levels and (**C**) concentrations of insulin after oral glucose gavage (2 g/kg body weight) in week 16 and corresponding incremental area under the curves (iAUC), n = 6. (**D**) Time-dependent BG levels after i.p. insulin (0.75 U/kg body weight) in week 16 and corresponding iAUC, n = 6. (**E**) Relative analysis of immunohistochemical (IHC)-stained insulin in whole pancreatic sections and representative images, n = 3. Data are mean +/− SEM, * *p* < 0.05. OGTT, oral glucose tolerance test; ITT, insulin tolerance test.

To determine whether C17:0 plays a causal role in insulin resistance, we performed different tolerance tests within our animal study. After 16 h of fasting, blood glucose and insulin concentrations were not different between HF-supplemented groups (Figure 3A). The OGTT also showed no differences in the blood glucose curve after the oral bolus (Figure 3B), but insulin concentrations were significantly different at the 15 min timepoint. Here, supplementation of C17:0 led to lower insulin secretion compared to HF control; however, this was not reflected in the incremental area under the curve (iAUC) including all timepoints (*p* > 0.99; Figure 3C). Supplementation of C17:0 also showed no difference in the ITT. Milk-fat-fed mice, however, demonstrated higher blood glucose concentrations after 60 min post insulin injection, compared to HF diet, which was also absent in the iAUC (Figure 3D). Overall, the data of the tolerance tests indicate that C17:0 supplementation has only a minor effect on insulin sensitivity under high-fat diet conditions, which is in line with the immunohistochemistry staining of insulin in the pancreas showing no differences between all HF groups (Figure 3E).

### 3.4. Supplementation of C17:0 Does Not Affect Hepatic Lipid Metabolism In Vivo

**Figure 4:**
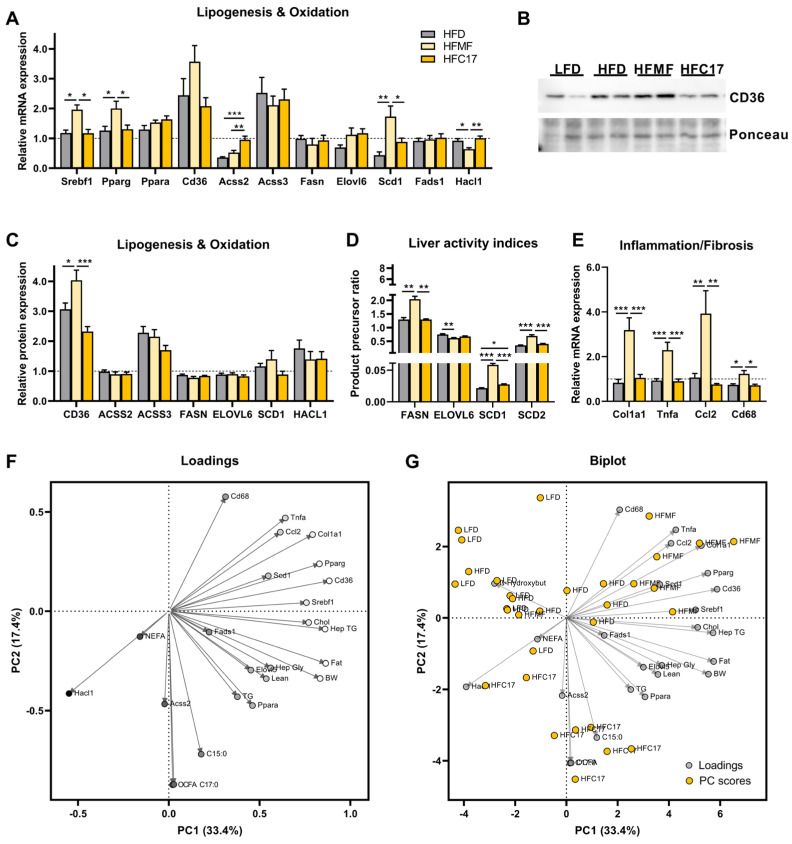
Long-term high-fat feeding with C17:0 has minor effects on hepatic lipid metabolism, while milk-fat-fed animals display pronounced signs of inflammation in the liver. C57BL/6JRj mice were fed semi-synthetic low-fat diet (LFD, dotted line), high-fat diet (HFD, depicted in grey) or HFD supplemented with either 14% milk fat (HFMF; depicted in light yellow) or 5% heptadecanoic acid (HFC17, depicted in dark yellow) for 20 weeks. (**A**) Relative hepatic expression of genes of lipogenesis and fatty acid oxidation. (**B**) Representative blot of CD36 with ponceau as loading control and (**C**) calculated relative protein expression of all detected proteins by Western blot, n = 6−8. (**D**) Calculated activity indices determined by the ratio of the product to precursor in fatty acid composition, n = 7−8. (**E**) Relative hepatic expression of genes of inflammation and fibrosis after 20 weeks of intervention, n = 7−8. Data were normalized to LFD = 1. Principal component (PC) analysis showing (**F**) the loading plot and (**G**) biplot of data including phenotypical characterization, OCFA formation and mRNA expression in the liver. Data are mean + SEM, * *p* < 0.05; ** *p* < 0.01, *** *p* < 0.001. OCFA, odd-chain fatty acids; FASN index, 16:0/18:2n6c; ELOVL6 index, 18:0/16:0; SCD1 index, 16:1n7c/16:0; SCD2 index, 18:1n9c/18:0.

Despite the increase in OCFA in both experimental diets, effects on liver lipid metabolism were markedly different between milk fat and C17:0 supplementation. Chronic dietary intake of milk fat showed a high induction of *Pparg* gene expression, compared to HF, but was unaffected by C17:0 (Figure 4A). Only after HFMF feeding was the expression of CD36, a fatty acid transporter and target of Pparg, significantly increased for both mRNA and protein levels (Figure 4A–C). Consistent with this, gene expression of several key inflammation and fibrosis markers (*Col1a1, Tnfa, Ccl2, Cd68*) was highly induced in the HFMF group (Figure 4E). Additionally, HFMF feeding increased gene expression of *Srebf1* (Figure 4A) and its targets in the de novo lipogenic pathway—evident by an increased hepatic expression of *Scd1* and by higher liver activity indices of FASN, SCD1 and SCD2 (Figure 4D), compared to HF and HFC17 diets. *Hacl1*, a key enzyme of α-oxidation, was only decreased with milk fat feeding, compared to HF, but remain unchanged after C17:0 supplementation (Figure 4A). Overall, C17:0 feeding exhibited no apparent effect on hepatic lipid metabolism in mice. Although gene expression of *Acss2* was increased by C17:0 (Figure 4A), protein expression was unaltered (Figure 4C), and no other enzymes were significantly affected after 20 weeks of feeding. Our data suggest that milk fat intake worsens the high-fat-diet-induced metabolic alterations, independently of C17:0.

Loadings and biplot were used to visualize patterns of correlation and clustering between all variables. The loadings plot showed that only *Hacl1* expression and to a lesser extent circulating NEFAs correlate negatively with PC1, whereas all other variables correlate positively (Figure 4F). In contrast, formation of OCFA was most negatively correlated with PC2. Together with the biplot, it was shown that HFMF mice have high values with the cluster of genes involved in inflammation/fibrosis as well as with the expression of *Srebf1, Pparg* and *Cd36*, indicating a relationship between these variables. The biplot further indicated that OCFA levels were dissimilar from measured variables, as reflected by separation of the data, and were more related to dietary intake of C17:0 (Figure 4G).

### 3.5. Hepatic Immune Signaling Is Diminished by OCFA Treatment In Vitro

**Figure 5:**
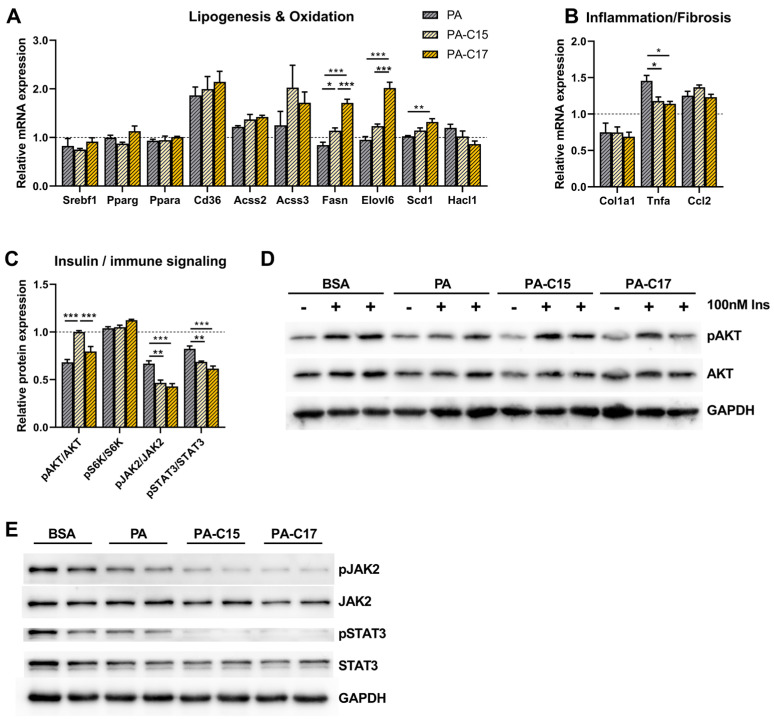
Treatment of primary hepatocytes with C17:0 has no impact on AKT but reduces JAK2-STAT3 signaling. mRNA expression analysis of genes involved in (**A**) fatty acid synthesis/oxidation and (**B**) inflammation/fibrosis of primary hepatocytes treated with BSA (dotted line, set to 1), palmitate control (PA; 250 µM), C15:0 (PA-C15; 125 µM PA:125 µM C15:0) or C17:0 (PA-C17; 125 µM PA:125 µM C17:0) for 24 h (n = 4). Protein expression analysis for proteins involved in (**C**) insulin signaling and (**D**) the representative blots of insulin-stimulated (100 nM Ins, 15 min) primary hepatocytes treated for 48 h with BSA, PA, C15:0 or C17:0. Protein expression analysis for proteins involved in (**C**) immune signaling and (**E**) the representative blots after primary hepatocytes were treated with BSA, PA, C15:0 or C17:0 for 48 h. (n = 4). Data are mean + SEM, * *p* < 0.05; ** *p* < 0.01, *** *p* < 0.001.

Cell culture experiments with primary hepatocytes were performed to discriminate between hepatic effects of the OCFA C15:0 and C17:0. Treatment with C17:0 mostly resulted in increased mRNA expression of lipogenic genes (*Fasn, Elovl6, Scd1*) compared to palmitate (PA) control and C15:0 (Figure 5A). Further, insulin stimulation of hepatocytes only with C15:0 led to an increased phosphorylation of AKT compared to PA and C17:0, while total AKT levels remained the same (Figure 5C,D). However, both OCFA show the same impact on inflammation signaling. Treatment of primary hepatocytes for 24 h with C15:0, as well as C17:0, reduced mRNA expression of *Tnfa*, a key cytokine in the inflammatory processes (Figure 5B). This was found together with significantly decreased phosphorylation levels of JAK2 and STAT3 after 48 h of stimulation with C15:0 and C17:0, compared to PA treatment alone (Figure 5C,E).

## 4. Discussion

Circulating concentrations of OCFA have been linked to health benefits in humans and rodents, but a mechanistic explanation behind these benefits is not clear. In this study, we show that a long-term intake of milk fat, which is rich in OCFA, or supplementation of C17:0 is not able to improve diet-induced hepatic steatosis or insulin resistance. Dietary milk fat actually exacerbates ectopic lipid accumulation and in parallel promotes the inflammatory state in the liver. However, we identified that treatment of primary hepatocytes with C15:0 and C17:0 suppresses JAK2/STAT3 signaling, which may indicate an anti-cancer and anti-NAFLD effect of OCFA.

Initially, the long-chain fatty acid profile in several compartments was measured to compare the induction of OCFA levels with the different dietary interventions. Changes in OCFA formation were mainly detected in liver and plasma phospholipids after intake of all dietary interventions. HFMF feeding only led to a significant induction of C15:0, while the total amount of OCFA was lower compared to C17:0 or propionate supplementation (also see Figure S1). Thus, these results indicate that OCFA are not suitable biomarkers for the intake of specific foods, such as dairy products. Our lab has already demonstrated that the intake of dietary fiber is also able to induce OCFA in humans and mice (through intestinal produced propionate), which is associated with a number of health benefits, such as the prevention of body weight gain, hepatic fat accumulation and insulin resistance in mice [21]. By comparing the amount of OCFA after dietary fiber or milk fat administration, it becomes clear that milk fat is more effective in inducing OCFA compared to dietary fiber, but the expected health benefits were absent.

A number of observational studies show an association between dairy fat consumption and multiple health outcomes [34,35,36]. Some have suggested that OCFA convey the positive effects of dairy fat [2]. In contrast, dietary guidelines often recommend to avoid dairy foods rich in fat, i.e., butter or cheese, as their intake can increase circulating cholesterol and drive CVD risk [37,38]. Despite higher levels of OCFA, this present study could show that HF feeding with milk fat supplementation neither prevents HF-induced body weight gain nor fat mass accumulation, nor does it affect glucose or insulin homeostasis in the long term. However, milk fat feeding drives rather detrimental effects on the hepatic phenotype, as triglycerides are higher, and intrahepatic lipid droplets are larger. Together, with an increased expression of markers for inflammation and fibrosis, the data provide evidence for an ongoing progression of lipotoxicity, which can induce ER stress and mitochondrial dysfunction and can further proceed to NASH development [39]. Likewise, we demonstrated that milk fat intake induces hepatic expression of *Pparg* as well as its downstream target CD36, a fatty acid transporter that is described as a driver for an exaggerated influx of fatty acids into hepatocytes, further triggering lipotoxicity effects [40]. The adverse effects in the liver may be exacerbated by the diet itself—milk fat also contains high amounts of saturated fatty acids (SFA), such as palmitic acid (C16:0) and stearic acid (C18:0), as depicted in Figure S5. Both SFA were reported to induce lipotoxicity via a JNK-dependent mechanism [41,42] and are described to be associated with enhanced serum total cholesterol in humans [43,44]. Naturally, milk fat already contains cholesterol [45]. Whether the increased circulating cholesterol after milk fat intake is dependent on cholesterol intake alone or may also be driven by higher SFA intake needs further investigation. It is likely that both together, the enhanced cholesterol and SFA levels, act synergistically to drive the worsening of the hepatic phenotype in the long term after high milk fat intake [46].

We also attempted to determine metabolic effects of the OCFA, heptadecanoic acid (C17:0), as it was found to have the strongest inverse association with incident type 2 diabetes in several studies [4,47]. In this study, supplementation of C17:0 to a high-fat diet elicited no differential effects on body weight gain or ectopic lipid accumulation in the liver after 20 weeks of feeding. This is in agreement with a recent publication showing that supplementation of oral C17:0 in obese mice for 90 days has no impact on body weight gain compared to non-supplemented controls. That study also shows that C15:0 supplemented mice had a lower percent body weight, lower cholesterol and lower circulating levels of proinflammatory cytokines after 12 weeks on a high-fat diet [20]. Within the same study, it was further clarified that daily oral C15:0 supplementation leads to lower triglycerides and less severe liver fibrosis in NASH-induced New Zealand white rabbits [20]. We found similar beneficial effects in mice fed a high-fat diet supplemented with propionate (see Figures S2–S4). This may suggest that propionyl-CoA is a common factor for both, since oxidation of OCFA as well as the catabolism of propionate leads to propionyl-CoA formation. However, next to the above-mentioned studies, a possible direct causal relationship between C17:0 and insulin sensitivity has not, to the best of our knowledge, been considered previously in rodent or human studies. The results presented here support a negligible effect of high-fat C17:0 supplementation on lowering insulin levels during an OGTT. Moreover, only treatment of primary hepatocytes with C15:0, and not of C17:0, showed an increased insulin-mediated phosphorylation of AKT, which plays a key role in intracellular insulin signaling and may suggest improved insulin sensitivity [48]. This is similar to results from an in vivo study showing that C15:0 promotes glucose uptake in myotubes via the AMPK pathway, indicating an insulin-sensitizing effect [49]. Together, the data support the fact that C17:0 supplementation does not attenuate HF diet-induced hepatic steatosis and insulin resistance. Although direct effects of C15:0 cannot be excluded in vivo here after HFC17 feeding, it appears that C17:0 does not play an important role in reported beneficial effects of dairy intake and OCFA, which are more likely to be mediated by other components or intermediates, e.g., milk protein, branched-chain amino acids or propionyl-CoA [21,27,31]. As propionyl-CoA is likely to be an important source of circulating OCFAs, the described beneficial association of plasma OCFAs could be attributed to enhanced propionyl-CoA availability. This is due to the fact that dietary propionate is sufficient to prevent high-fat-diet-induced hepatic steatosis and insulin resistance, partly explained by reduced hepatic fatty acid transport (see Figures S2–S4).

Recently, Venn-Watson et al. proposed C15:0 as an essential fatty acid because of its broad anti-inflammatory and antiproliferative activities in vivo and in vitro [20,50,51]. Further, it has been explored that C15:0 has an inhibitory effect on the IL-6-induced JAK2/STAT3 signaling pathway in human breast carcinoma cells, considering C15:0 as a promising therapeutic strategy to treat human cancers [17,18]. Little has been described with C17:0 in this context—only one study has focused on the role of C17:0 in lung cancer cells, where they showed inhibited cell proliferation and a greater promotion of apoptosis in C17:0-treated lung adenocarcinoma cells [23]. Results of our cell culture experiments imply that both C15:0 and C17:0 are similarly able to diminish proinflammatory signaling in hepatocytes. A lower gene expression of *Tnfa*, which is a key proinflammatory cytokine [52], further leads to a reduced phosphorylation of JAK2 and STAT3 after C15:0 and C17:0 treatment. Unfortunately, the data of our in vivo study do not support an anti-inflammatory effect of C17:0 in the liver, although others reported reduced levels of the circulating proinflammatory chemokine MCP-1 in C17:0-supplemented mice compared to controls [20]. From the current study, it is still unclear whether both OCFA are effective mediators in the suppression of the hepatic JAK2/STAT3 signaling pathway in vivo, but the data presented here are promising for ongoing studies.

In conclusion, our data provide new evidence that the published beneficial association of plasma OCFA and a decreased type 2 diabetes risk are not mediated by C17:0. Milk fat intake, as an exogenous source of OCFA, even facilitates ectopic lipid accumulation and inflammation in the liver. This emphasizes the possibility that other factors, such as C15:0 levels or propionyl-CoA availability, are more likely to contribute to improved metabolic health, which require further investigation.

## Data Availability

The data presented in these studies are available on request from the corresponding author.

## Supplementary Materials

The following supporting information can be downloaded at: https://www.mdpi.com/article/10.3390/nu15092052/s1, Figure S1: Supplementation of propionate leads to an induction of hepatic and circulating OCFA; Figure S2: High-fat feeding with propionate inhibits excessive hepatic lipid accumulation; Figure S3: Long-term supplementation of propionate improves insulin sensitivity; Figure S4: Long-term high-fat feeding with propionate results in a diminished hepatic fatty acid transport; Figure S5: Fatty acid composition of diets used in this study, measured by gas chromatography; Table S1: Composition of experimental semi-synthetic diets used in this study; Table S2: Oligonucleotides used in this study to measure mRNA levels.
