# Supplementary material for: Heptadecanoic Acid Is Not a Key Mediator in the Prevention of Diet-Induced Hepatic Steatosis and Insulin Resistance in Mice
Source: Nutrients. 2023 Apr 24;15(9):2052. doi: 10.3390/nu15092052 (PMC10180762; doi:10.3390/nu15092052)
Supplement: Supplementary file 1 [file nutrients-15-02052-s001.zip › Supplementary Files_Tables.pdf]

Supplementary Files

Table S1: Composition of experimental semi-synthetic diets used in this study.

**Components**	**LFD (g/kg)**	**HFD (g/kg)**	**HFMF (g/kg)**	**HFC17 (g/kg)**	**HFPr (g/kg)**
Casein	140	140	140	140	140
Wheat starch	467	300	300	300	300
Maltodextrin	100	100	100	100	100
Dextrose	50	50	50	50	50
Sucrose	100	100	100	100	100
Lipids [1]	43	210	70	160	145
Cellulose	50	50	50	50	50
**Milk fat**			**140**		
**C17:0**				**50**	
**Sodium propionate**					**65**
Mineral mixture	35	35	35	35	35
Vitamin mixture	10	10	10	10	10
Choline bitartrate	2.5	2.5	2.5	2.5	2.5
L-Cysteine	3	3	3	3	3
Measured energy content (kJ/g) [2]	16.3	19.4	19.9	19.5	18.6

[1] Composed of 70% sunflower oil, 18% coconut oil and 12% flaxseed oil.
[2] Measured by bomb calorimetry.

Table S2: Oligonucleotides used in this study to measure mRNA levels.

**Primer name**		**Primer sequence (5′→3′)**
B2m	f	CCC CAC TGA GAC TGA TAC ATA CGC
	r	AGA AAC TGG ATT TGT AAT TAA GCA GGT TC
Srebf1	f	GAG GAT AGC CAG GTC AAA GC
	r	AGG ATT GCA GGT CAG ACA CA
Pparg	f	TGC CAA AAA TAT CCC TGG TT
	r	GGC GGT CTC CAC TGA GAA TA
Ppara	f	ATT CGG CTG AAG CTG GTG TA
	r	AAG CGA ATT GCA TTG TGT GA
Cd36	f	CCA AGC TAT TGC GAC ATG AT
	r	ACA GCG TAG ATA GAC CTG CAA A
Acss2	f	TGT GTG ATG GGC CAT ACC TTC

	r	GTA GTC TGG TGT GGC AAT GG
Acss3	f	ACC AGG AAG GAA GGT GGA GT
	r	AAC TCT GTC TGG CCT GTG CT
Fasn	f	TTG ATG ATT CAG GGA GTG GA
	r	TTA CAC CTT GCT CCT TGC TG
Elovl6	f	TGC AGG AAA ACT GGA AGA AGT CT
	r	AGC GGC TTC CGA AGT TCA A
Scd1	f	TTC TTC TCT CAC GTG GGT TG
	r	CGG GCT TGT AGT ACC TCC TC
Fads1	f	CTC GTG ATC GAC CGG AAG GT
	r	CCA CAA AAG GAT CCG TGG CAT
Hacl1	f	ACA GGC TTG ATG CAG GTT CT
	r	CCA CTT CCA TGC CAG AAA AT
Col1a1	f	GTG CTC CTG GTA TTG CTG GT
	r	GGC TCC TCG TTT TCC TTC TT
Tnfa	f	CCA CCA CGC TCT TCT GTC T
	r	GCT CCT CCA CTT GGT GGT TT
Ccl2	f	CAC TCA CCT GCT GCT ACT CA
	r	GCT TGG TGA CAA AAA CTA CAG C
Cd68	f	GGA CTA CAT GGC GGT GGA ATA C
	r	GAG AGC AGG TCA AGG TGA ACA G
